# Apigenin attenuates LPS-induced neurotoxicity and cognitive impairment in mice via promoting mitochondrial fusion/mitophagy: role of SIRT3/PINK1/Parkin pathway

**DOI:** 10.1007/s00213-022-06262-x

**Published:** 2022-10-26

**Authors:** Omaima A. Ahmedy, Tarek M. Abdelghany, Marwa E. A. El-Shamarka, Mohamed A. Khattab, Dalia M. El-Tanbouly

**Affiliations:** 1 Department of Pharmacology and Toxicology, Faculty of Pharmacy, Cairo University, Cairo, 11562 Egypt; 2 School of Biomedical, Nutritional and Sport Sciences, Faculty of Medical Sciences, Newcastle University, Newcastle Upon Tyne, NE24HH UK; 3 Institute of Translational and Clinical Research, Newcastle University, Newcastle Upon Tyne, NE24HH UK; 4 Department of Narcotics, Ergogenic Aids and Poisons, Medical Research Division, National Research Center, Cairo, 12622 Egypt; 5 Department of Cytology and Histology, Faculty of Veterinary Medicine, Cairo University, Cairo, 12211 Egypt

**Keywords:** NAD^+^, SIRT3, Apigenin, Mitochondrial fusion, Mitophagy, ATP

## Abstract

**Rationale:**

Alteration of the NAD^+^ metabolic pathway is proposed to be implicated in lipopolysaccharide (LPS)-induced neurotoxicity and mitochondrial dysfunction in neurodegenerative diseases. Apigenin, a naturally-occurring flavonoid, has been reported to maintain NAD^+^ levels and to preserve various metabolic functions.

**Objectives:**

This study aimed to explore the effect of apigenin on mitochondrial SIRT3 activity as a mediator through which it could modulate mitochondrial quality control and to protect against intracerebrovascular ICV/LPS-induced neurotoxicity.

**Methods:**

Mice received apigenin (40 mg/kg; p.o) for 7 consecutive days. One hour after the last dose, LPS (12 µg/kg, icv) was administered.

**Results:**

Apigenin robustly guarded against neuronal degenerative changes and maintained a normal count of intact neurons in mice hippocampi. Consequently, it inhibited the deleterious effect of LPS on cognitive functions. Apigenin was effective in preserving the NAD^+^/NADH ratio to boost mitochondrial sirtuin-3 (SIRT3), activity, and ATP production. It conserved normal mitochondrial features via induction of the master regulator of mitochondrial biogenesis, peroxisome proliferator-activated receptor γ (PPARγ) coactivator-1α (PGC-1α), along with mitochondrial transcription factor A (TFAM) and the fusion proteins, mitofusin 2 (MFN2), and optic atrophy-1 (OPA1). Furthermore, it increased phosphatase and tensin homolog (PTEN)-induced putative kinase 1 (PINK1) and parkin expression as well as the microtubule-associated protein 1 light chain 3 II/I ratio (LC3II/I) to induce degradation of unhealthy mitochondria via mitophagy.

**Conclusions:**

These observations reveal the marked neuroprotective potential of apigenin against LPS-induced neurotoxicity through inhibition of NAD^+^ depletion and activation of SIRT3 to maintain adequate mitochondrial homeostasis and function.

**Graphical abstract:**

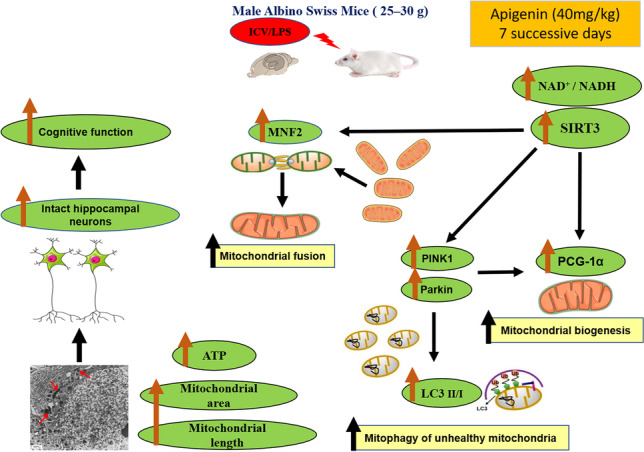

## Introduction

Mitochondria have been gaining increasing attention as the likely target for numerous neurotoxins. They are crucial and highly dynamic organelles for neuronal function and survival (Bertholet et al. 2016). Their presence in the axonal ends and nodes of Ranvier plays a vital role in ensuring adequate energy supply to neurons as well as functional axonal transport by facilitating appropriate Ca^2+^ homeostasis (Giorgi et al. 2018). However, mitochondria could not exert their function without the essential dynamic balance between mitochondrial fusion and fission, along with the selective elimination of damaged mitochondria through effective mitophagy. These processes are essential for effective mitochondrial quality control, which in turn impacts on normal mitochondrial function and maintains their morphology under altering physiological conditions (Meng et al. 2019; Zhu et al. 2013).

Contrary to mitochondrial fission, mitochondrial fusion ensures the completion of mitochondrial genomic (mtDNA) required for oxidative phosphorylation. Fused mitochondrial networks are important for energy transmission between different neuronal regions (Ibáñez et al. 2015; Vásquez-Trincado et al. 2016). Mitochondrial fusion is a two-step process that necessitates the fusion of the outer and inner mitochondrial membranes, respectively, through the action of the mitofusin1/2 (MFN1, MFN2) and ocular atrophy 1 (OPA1). OPA1 simultaneously governs mitochondrial fusion while preserving proper oxidative phosphorylation, as shown by its control over the morphology of the mitochondria’s cristae following fusion, which directly impacts the stability of electron transport chain complexes. Loss of any of the aforementioned proteins gives rise to small fragmented mitochondria resulting in the potential loss of the mitochondrial membrane and reduced respiration (Meng et al. 2019). Furthermore, mitochondrial fusion enhances the turnover of injured mitochondria by selective mitophagy (Maneechote et al. 2017; Zhou et al. 2018).

Numerous mitophagy pathways have been recognized, of which the PINK1-Parkin pathway is the most extensively studied (Ziviani and Whitworth 2010). Under normal conditions, low levels of phosphatase and tensin homolog (PTEN)-induced putative kinase 1 (PINK1) are maintained through complex processing. With decreased mitochondrial membrane potential (MMP) in damaged mitochondria, PINK1 is stabilized on the outer mitochondrial membrane (OMM), with the consequent recruitment of Parkin, the E3 ubiquitin ligase. The latter translocates to the mitochondria from the cytosol, targeting several mitochondrial membrane proteins to instigate the degradation process (Narendra et al. 2008). Finally, mitophagy is induced by the recruitment of microtubule-associated protein 1 light chain 3 (LC3) adaptors to the OMM, which are then recognized by LC3 and facilitate the production of autophagosomes (Lazarou et al. 2015).

Mitochondrial mitophagy and biogenesis are two opposite interplaying processes that play a pivotal role in governing cell fate in cells such as neurons (Zhu et al. 2013). Peroxisome proliferator-activated receptor γ (PPARγ) coactivator-1α (PCG-1α) is the main mitochondrial biogenesis regulator to generate new mitochondria. It regulates the activity of mitochondrial transcription factor A (TFAM), a crucial activator of mtDNA transcription and replication of the mitochondrial genome (Uittenbogaard and Chiaramello 2014). The mitochondrial NAD^+^-dependent deacetylase sirtuin-3 (SIRT3) is considered one of the most influential mitochondrial deacetylases involved in the regulation of mitochondrial function, biogenesis, and quality control in neurons (Sidorova-Darmos et al. 2018). SIRT3 activity relies on NAD^+^ levels, whose depletion induces neuronal death and accelerates aging (Satoh et al. 2017; Lautrup et al. 2019). Along with sirtuins, NAD^+^ is a substrate for various proteins such as CD38, the primary NAD^+^ase in mammalian cells (Chini et al. 2018). It has been reported that the age-dependent rise in CD38 activity contributes to NAD^+^ diminution and impaired mitochondrial function (Camacho-Pereira et al. 2016).

The bacterial endotoxin, lipopolysaccharide (LPS), is an essential element in the gram-negative bacterial cell wall (Biesmans et al. 2013). Various studies have reported that LPS induces neurotoxicity via stimulating immune cells, including brain glial cells (Johansson et al. 2005; Khan et al. 2018). Nevertheless, impairing the key bioenergetic role of mitochondria is considered the main mechanism of neurotoxicity, where dysfunction of mitochondrial homeostasis has been broadly associated with neuronal degeneration (Johri and Beal 2012). Thus, the emerging importance of the interplay between mitochondrial dynamic, biogenesis, and autophagy signaling pathways sheds light on therapeutic approaches targeting or regulating mitochondrial homeostasis in response to neurotoxins.

Apigenin, a polyphenolic flavonoid, possesses a range of biological activities including antioxidant, anti-inflammatory, anticancer, and antidiabetic effects (Salehi et al. 2019; Yammine et al. 2020). Furthermore, apigenin supplementation was reported to exert therapeutic effects on Alzheimer’s disease (AD), insomnia, anxiety disorder, and depression in humans (Salehi et al. 2019). Increasing evidence has shown that apigenin inhibits CD38 activity, maintaining NAD^+^ levels, promoting lipid and glucose homeostasis, and exerting nephroprotective effects in obese animals (Escande et al. 2013; Ogura et al. 2020).

Therefore, the current study was directed to explore the possible effect (s) of apigenin on SIRT3 activity as a mechanistic mediator through which it could modulate several components of the regulatory machinery of mitochondrial quality control to protect against ICV-LPS-induced hippocampal neurotoxicity and cognitive impairment in mice.

## Materials and methods

### Animals

Swiss albino male mice (8 weeks old) weighing 25–30 g were procured from the Faculty of Veterinary Medicine, Cairo University, Egypt. Animals were housed in groups of 6 per cage and acclimatized for 1 week before the study commencement and kept at a temperature of (22 ± 3 °C) with constant relative humidity and an alternate 12 h light/dark cycle in the Faculty of Pharmacy Animal Facility, Cairo University. Mice were provided a standard rodent chow diet and free access to water. All procedures were approved by the institutional ethics committee for the care and use of animals in accordance with the ethics of *The Guide for Care and Use of Laboratory Animals* (NIH Publication No. 8523, revised 2011) and accepted by the Research Ethical Committee of the Faculty of Pharmacy (PT 2768).

### Drugs and chemicals

LPS from Escherichia coli O111:B4 (Cat. # L4391) and apigenin (Cat. # 10,798) were procured from Sigma-Aldrich (St Louis, USA).

### Induction of neurotoxicity

Neurotoxicity was induced by the intracerebroventricular (ICV) injection of LPS. The modified ICV procedure in mice ((Rasheed et al. 2018; Pelleymounter et al. 2000, 2002) was employed to avoid the induction of physiological stress and prevent any possible penetration into the cerebral vein (McConn et al. 2015, 2019). Animals were anesthetized with thiopental (5 mg/kg) (Wadie and El-Tanbouly 2017) and the head was fixed using light pressure applied above the ears. A 30-gauge needle was introduced perpendicularly into the lateral ventricle through the “soft spot” where the lateral sutures of the skull bones and the midline intersect. The site of injection was located approximately 1 mm into the midline point to an equal distance between the eyes and ears, 0.8 mm posterior to the bregma, 1.0 mm proximate to the sagittal suture, and 3.0 mm through the dorsal plane of the skull underneath the brain surface (Fronza et al. 2019; Ashour et al. 2021).

### Experimental design

Seventy-two mice were allocated into four groups, each of 18 mice: group 1 received normal saline ICV (normal control), group 2 received apigenin (40 mg/kg) (Zhao et al. 2013) orally for 7 consecutive days (ICV-saline/apigenin), group 3 received a single injection of LPS (12 µg/kg, icv) (Zhao et al. 2019) (ICV-LPS), group 4 received apigenin orally for 7 consecutive days and on the 7th day, 1 h after the last apigenin dose, LPS (12 µg/kg, icv) was administered (ICV-LPS/apigenin). The Y maze task was used to assess animal behavior 4 h after ICV-LPS injection (Zhou et al. 2006) followed by the Morris Water Maze (MWM), starting with the least stressful activity and progressing to the most stressful one, with a 30-min rest in between each task’s intensity. Then, mice were sacrificed by decapitation under light anesthesia and their brains were removed. For histopathological examination, the brains of 4 randomly selected mice from each group were preserved in formalin (10%) for 24 h. Hippocampi from 4 mice were isolated and immersed in cold glutaraldehyde (2.5%) with cacodylate buffer (0.1 mol/L, pH 7.4), to perform an electron microscopy examination. The hippocampi of 6 animals were homogenized to obtain a 10% homogenate in cold phosphate buffer saline (PBS; pH = 7.4). The hippocampi of the remaining animals were stored at − 80 °C to be used for the Western blotting technique.

### Behavioral assessments

#### Morris water maze (MWM) test

Morris water maze, a commonly approved method of visuospatial memory and learning capacity testing, was performed as described previously (D’Hooge and De Deyn 2001). A large circular tank (150 cm diameter, 60 cm height) was utilized. Using two threads fixed to the edge of the tank and perpendicular to each other, the tank was arbitrarily partitioned into four equal quadrants (NE, SE, NW, and SW). The tank was half-filled with tap water, rendered opaque with a purple dye, and kept at 23 ± 2 °C. In the target quadrant, a black-painted submerged platform (10 cm in diameter, 28 cm in height) was immersed and fixed 2 cm beneath the water surface. The escape platform location was kept fixed and constant during the test. Under normal circumstances, mice easily learn to swim directly in the direction of the escape platform and reach it quickly. Following the method of Gupta and Gupta (2012), all animals were trained from day 3 to day 6 before LPS administration, and each mouse was subjected to four training trials per day. Mice were introduced to the pool from random starting points, and the maximum time to detect the hidden platform was 120 s for each trial, and the mean escape latency (MEL) was recorded on each training day. The beginning positions (NE, N, W, and SW) for the mouse were chosen based on their nearly equal distances from the platform. During training days, starting positions were randomly chosen for each trial and their order was altered. The animal that failed to reach the escape platform during the nominated time was directed toward the platform and remained there for 20 s. On the 7th day, each animal was permitted to explore the tank for 60 s after the platform removal. To assess the ability of mice to retrieve their memory, the time spent in the target quadrant by each mouse was recorded (Blokland et al. 2004).

#### Y-maze test

A three-armed Y-shaped metallic maze was used to evaluate the short-term memory in the present investigation (Luszczki et al. 2005). The arms (10 cm wide, 25 cm high, 35 cm long) extended from a central platform and were positioned at 120°. Normally, animals tend to visit a new arm rather than the formerly explored arm. On the test day of the present study, the sequence and order of entries were recorded during a session of 8 min for each animal. After every session, the maze’s arms were cleaned with 70% ethanol to eliminate any olfactory signals resulting in incorrect observations. The overlapping triplet sets, which are defined by the successive entries into all the Y-maze’s arms, were recorded. The total number of arms entries minus two represented the possible alternations. The percentage of spontaneous alternation was obtained by dividing actual alternations by the number of possible alternations X 100 (Yamada et al. 1999).

### Mitochondrial separation

Tissue homogenates were centrifuged at 4 °C for 10 min with 0.25 M sucrose at 2000 g. The supernatants were mixed with 0.75 M sucrose in 4-(2-hydroxyethyl)-1-piperazineethanesulfonic acid (HEPES) buffer and centrifuged for 30 min at 10,000 g. The supernatants were then discarded; the pellets were re-suspended in HEPES buffer and re-centrifuged at 10,000 g for 10 min. The washed pellets enriched with mitochondria were then re-suspended in PBS and stored in aliquots at − 80 °C until required.

## Assessed parameters

### Western blot analysis

Fusion markers (MFN2 and OPA1), biogenesis markers (PCG-1α and TFAM), and mitophagy markers (PINK 1, Parkin, LC3I, and LC3II) expressions were determined using the Western blot technique. Twenty μg of protein from each sample were loaded well. The protein bands from the gel were transferred onto polyvinylidene difluoride membranes obtained from Thermo Fischer Scientific, MA, USA, by their assembly in a transfer sandwich using BioRad Trans-Blot Turbo for 7 min. Following blot transfer, a solution of 20 mM buffer here? pH 7.5, 0.1% Tween 20, 150 mM NaCl, and 3% bovine serum albumin and was used to block the membrane for 1 h at room temperature. Then, incubation of the membrane with the targeted antibodies was done at 4 °C overnight. The used antibodies were anti-MFN2 (PA5-42,171), anti-OPA1 (Cat. # PA5-98,029), anti- PGC-1α (Cat. # PA5-72,948), anti-TFAM (Cat. # PA5-68,789), anti-anti-PINK1 (Cat. # PA5-23,072), anti-Parkin (Cat. # 39–0900), anti-LC3I/LC3II polyclonal antibodies (Cat. # PA1-16,931), or anti- beta-actin (β-actin) (Cat # MA5-15,739) (Thermo Fischer Scientific, MA, USA). Subsequently, the blot was washed with TBST several times for 5 min. The blot was incubated with the HRP-conjugated secondary antibodies (Thermofisher Scientific, USA, Cat. # 31,460) at room temperature for 60 min. after that, the rinsing process was repeated. CCD camera-based imager was used to capture the chemiluminescent signals and a Chemi Doc MP imager (Bio-Rad, USA) was used to detect the target protein band related to the housekeeping protein β-actin.

### Enzyme-linked immunosorbent assay

MFN2 and PGC-1α biomarkers were determined also using Mouse ELISA Kits (Cat. # MBS9717649) and (Cat. # MBS1601099), respectively supplied by MyBioSource, Inc., San Diego, CA, USA. The assessed parameters were performed following the protocol provided by the manufacturer. The protein concentration of each sample was assessed using a Bradford protein assay kit (Bio basic, Canada, Cat.# SK3041).

### Determination of SIRT3 activity

Mitochondrial SIRT3 activity was assessed using fluorometric kits (Cyclex, Japan, Cat# CY-1153V2) according to the manufacturer’s instructions.

### Determination of NAD+/NADH

To assess the energy transformation and redox state of the tissue, nicotinamide nucleotides were determined. BioVision’s NAD^+^/NADH quantification kit (Milpitas, CA, USA: Cat. # K337-100) was used to detect the intracellular nucleotides: NAD^+^, NADH, and their ratio.

### Determination of ATP content

ATP, as a marker of mitochondrial function, was quantified in hippocampal tissues using a specific colorimetric assay kit (BioVision, Milpitas, CA USA, Cat# K354-100). The method exploits the phosphorylation of glycerol to produce a product that is quantified colorimetrically at 570 nm, according to the manufacturer’s instructions.

### Transmission electron microscopy

Sampled brain hemisphere was harvested and then the hippocampal area was immediately dissected and immersed in (2.5%) cold glutaraldehyde with cacodylate buffer (0.1 mol/L; pH 7.4), postfixed in 1% OsO4, dehydrated, and embedded in Epoxy resin. Regardless of the orientation, 80-μm ultrathin serial sections were cut and mounted on copper grids and stained with lead citrate and uranyl acetate. Sections were then examined and imaged using a JEOL transmission electron microscope (JEM-1400 TEM, Peabody, MA, USA) at the transmission electron microscopy laboratory, Faculty of Agriculture, Cairo University Central Research Laboratory. Six random non-overlapping electron microscopic fields were selected for measuring the mean mitochondrial length as well as the mean area percentage of manually colored and segmented mitochondria relative to the total electron microscopic field area.

### Histological examination

Brain samples were fixed for 72 h in neutral buffered formalin (10%). The samples were trimmed and processed using serial grades of ethanol. They were embedded into Paraplast tissue-embedding media after being cleared in Xylene. Using a rotatory microtome, 4-μm-thick serial sagittal brain sections were cut for demonstration of hippocampal regions in different samples and mounted on glass slides. Hematoxylin and Eosin staining was used as a general morphological investigation staining method. Additionally, sections stained with toluidine blue showing selective staining of Nissl granules were used for the detection of intact and damaged neurons. Six non-overlapping fields were randomly chosen and examined from the CA1 and CA3 hippocampal regions of each sample to count the number of intact neurons. A full HD microscopic imaging system was used to obtain all light microscopic examination and data (Leica Microsystems GmbH, Germany). Each mouse was given a score between 0 and 4 for each of two parameters: neuronal damage and edema, where, 0 indicates no lesions, 1 indicates few lesions in one examined section, 2 indicates mild lesions were focally demonstrated in some examined sections, 3 indicates moderate lesions were diffusely demonstrated in some examined sections, and 4 indicates severe lesions were diffused in all examined sections. By summing the two criteria for each mouse, a total histology score of up to 8 was determined.

### Data and statistical analysis

Homogeneity of variance and normality of distribution were verified using the Brown-Forsythe and Shapiro–Wilk tests, respectively. The values are expressed as mean ± S.D. Tukey’s multiple comparison test was used as a post hoc test after results were analyzed using two-way ANOVA, except for the total histopathological score which was expressed as median and analyzed using the Kruskal–Wallis test followed by Dunn’s test. Statistical significance was accepted at *p* < 0.05. All the statistical analyses were run utilizing GraphPad Prism software version 9 (CA, USA).

## Results

Animals that received apigenin alone showed an insignificant difference in all tested parameters except for MFN2, PGC-1 α, and SIRT3 activity as compared to the normal untreated group.

### Apigenin attenuated ICV-LPS-induced cognitive impairment

**Fig. 1:**
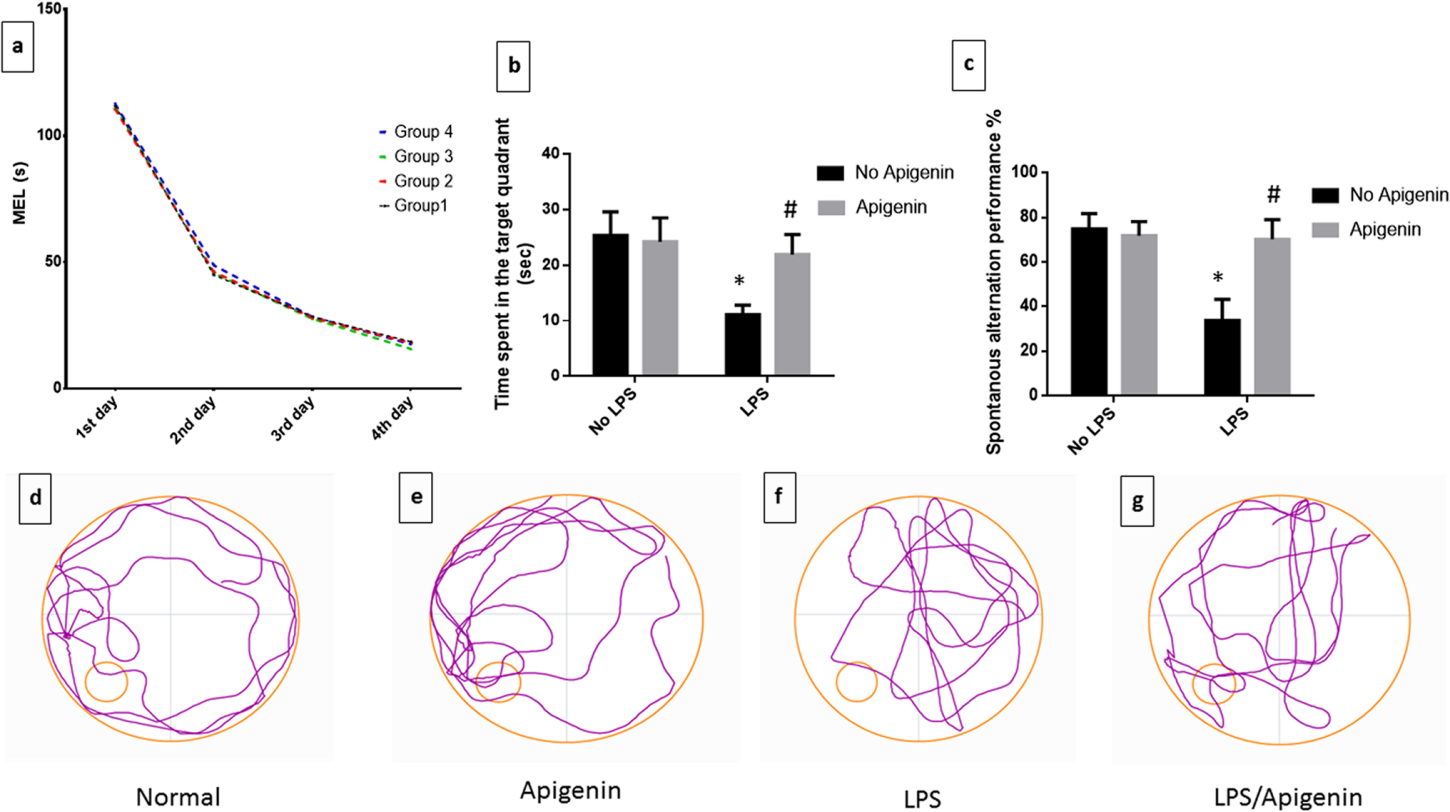
Effect of apigenin on ICV-LPS-induced cognitive dysfunction. **a** MEL during different training days before LPS administration, **b** time spent in the target quadrant in MWM on the probe day, **c** spontaneous alternation performance in the Y maze task, and **d–g** representative images of mouse track trail in the MWM during the probe day form each group. Each bar with a vertical line represents the mean ± S.D (*n* = 15). Statistically significant differences (< 0.05) from the normal control group and from the ICV-LPS group are denoted by * and # respectively using two-way ANOVA followed by Tukey’s multi-comparison test

In the MWM task, MEL has not shown any significant difference between the tested groups on each training day before LPS administration [*F* (3, 224) = 0.7189, *p* = 0.5416]. A significant difference was observed in MEL in the four groups between the training days, where mice exhibited a significant decrease in MEL in each subsequent training day as compared to the preceding one indicating normal acquisition [*F* (3, 224) = 2482, *p* < 0.0001]. Accordingly, no interaction was recorded between the two factors (treatment and different training days) [*F* (9, 224) = 0.3632, *p* = 0.9514] (Fig. 1a).

ICV-LPS injection resulted in a decline in cognitive function. Mice that received LPS exhibited a profound reduction in time spent in the target quadrant to 44.5% [*F* (1, 56) = 79.43, *p* < 0.0001] and spontaneous alternation performance to 45% [*F* (1, 56) = 107.8, *p* < 0.0001] relative to the normal group. On the other hand, apigenin effectively prolonged the time spent in the target quadrant to 86.3% [*F* (1, 56) = 26.78, *p* < 0.0001] and increased spontaneous alternation performance to 93.7% [*F* (1, 56) = 65.98, *p* < 0.0001] showing similar results as those of normal mice. For the two measured parameters interactions were recorded between the two factors (LPS and apigenin) [*F* (1,56) = 41.85 and 91.89, respectively, *p* < 0.0001] (Fig. 1b and c).

### Apigenin inhibited ICV-LPS-induced alterations in hippocampal ATP, mitochondrial biogenesis, and fusion markers

LPS-challenged mice demonstrated a significant diminution in hippocampal ATP [*F* (1, 20) = 107.0, *p* < 0.0001], PGC-1α as measured by Elisa [*F* (1, 20) = 83.77, *p* < 0.0001] as well as by WB [*F* (1, 12) = 83.77, *p* < 0.0001] and TFAM [*F* (1, 12) = 333.9, *p* < 0.0001].

**Fig. 2:**
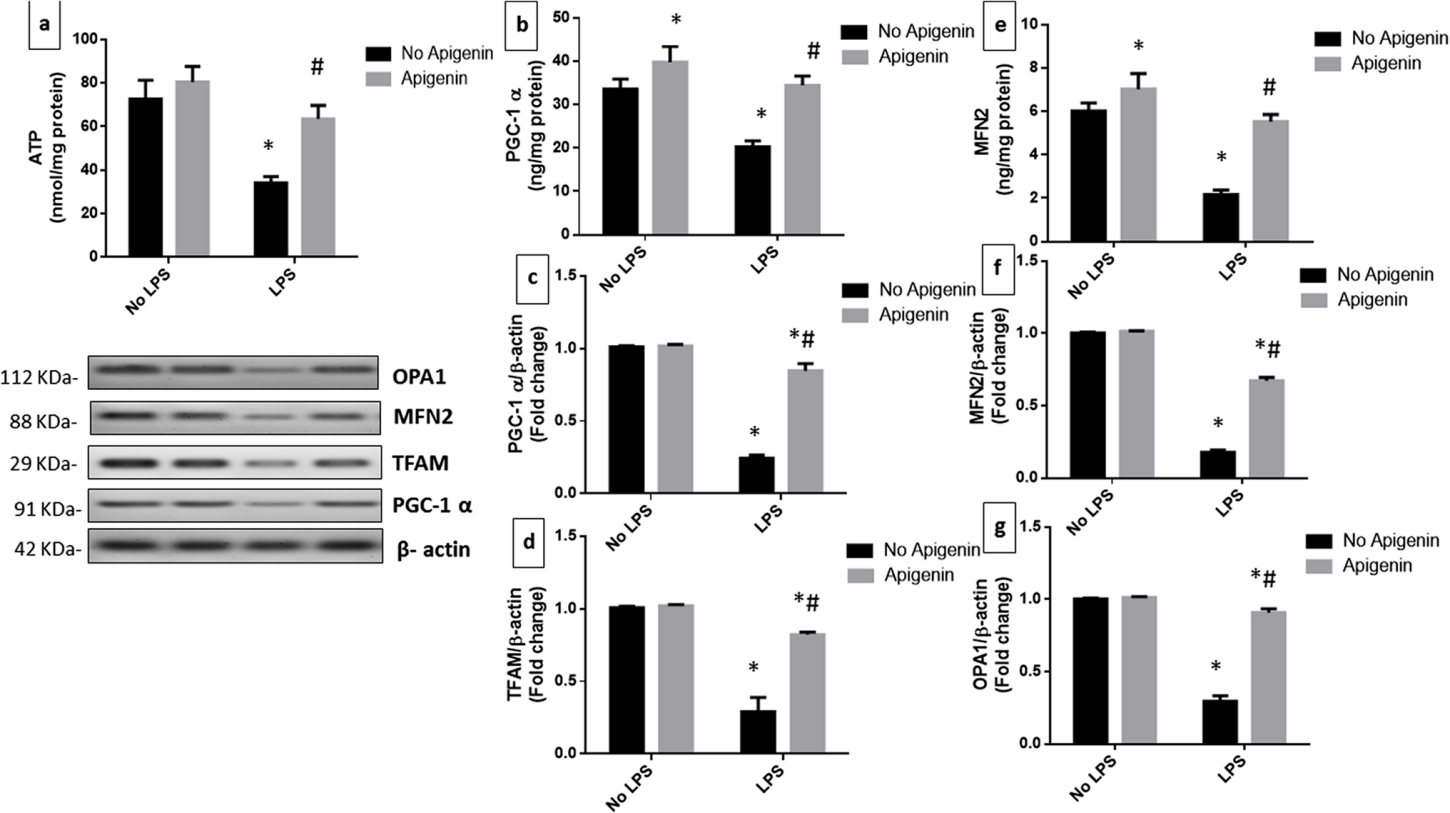
Effect of apigenin on ICV-LPS-induced alterations in **a** hippocampal ATP, **b** mitochondrial PGC-1α (Elisa), **c** PGC-1α (WB), **d** TFAM, **e** MFN2 (Elisa), **f** MFN2 (WB), and **g** OPA1. Each bar with a vertical line represents the mean ± S.D (*n* = 6 and 4 for WB). Statistically significant differences (< 0.05) from the normal control group and from the ICV-LPS group are denoted by * and # respectively using two-way ANOVA followed by Tukey’s multi-comparisons test

Apigenin effectively abrogated the loss of ATP levels [*F* (1, 20) = 48.59, *p* < 0.0001] and enhanced PGC-1α as measured by Elisa [*F* (1, 20) = 99.38, *p* < 0.0001] as well as by WB [*F* (1, 12) = 494.1, *p* < 0.0001] and TFAM [*F* (1, 12) = 118.5, *p* < 0.0001], to reach 1.7-, 3.5-, and 2.8-fold greater values than those measured in the ICV-LPS group. Interactions were recorded between the two factors (LPS and apigenin) for ATP [*F* (1, 20) = 16.27, *p* < 0.001], for PGC-1α [*F* (1, 20) = 15.19, *p* < 0.001 and *F* (1,12) = 478, *p* < 0.0001] and for TFAM [*F* (1, 12) = 106.8, *p* < 0.0001] (Fig. 2a–d).

In addition, hippocampal MFN2 was markedly decreased following LPS administration as measured by Elisa [*F* (1, 20) = 208.2, *p* < 0.0001] as well as by WB [*F* (1, 12) = 6854, *p* < 0.0001], along with OPA1 [*F* (1, 12) = 1154, *p* < 0.0001] relative to normal mice. In a similar fashion, apigenin was effective in increasing MFN2 as measured by Elisa [*F* (1, 20) = 138.7, *p* < 0.0001] as well as by WB [*F* (1, 12) = 1292, *p* < 0.0001], along with OPA1 [*F* (1, 12) = 672,2, *p* < 0.0001] to reach 2.6-, 3.8-, and 3.1-fold greater values than those measured in the ICV-LPS group. Interactions were recorded between the two factors (LPS and apigenin) for MFN2 ([*F* (1, 20) = 40.6, *p* < 0.0001 and *F* (1, 12) = 1192, *p* < 0.0001] and for OPA1 [*F* (1, 12) = 640, *p* < 0.0001] (Fig. 2e–g).

### Apigenin attenuated ICV-LPS-induced alterations in mitophagy parameters

**Fig. 3:**
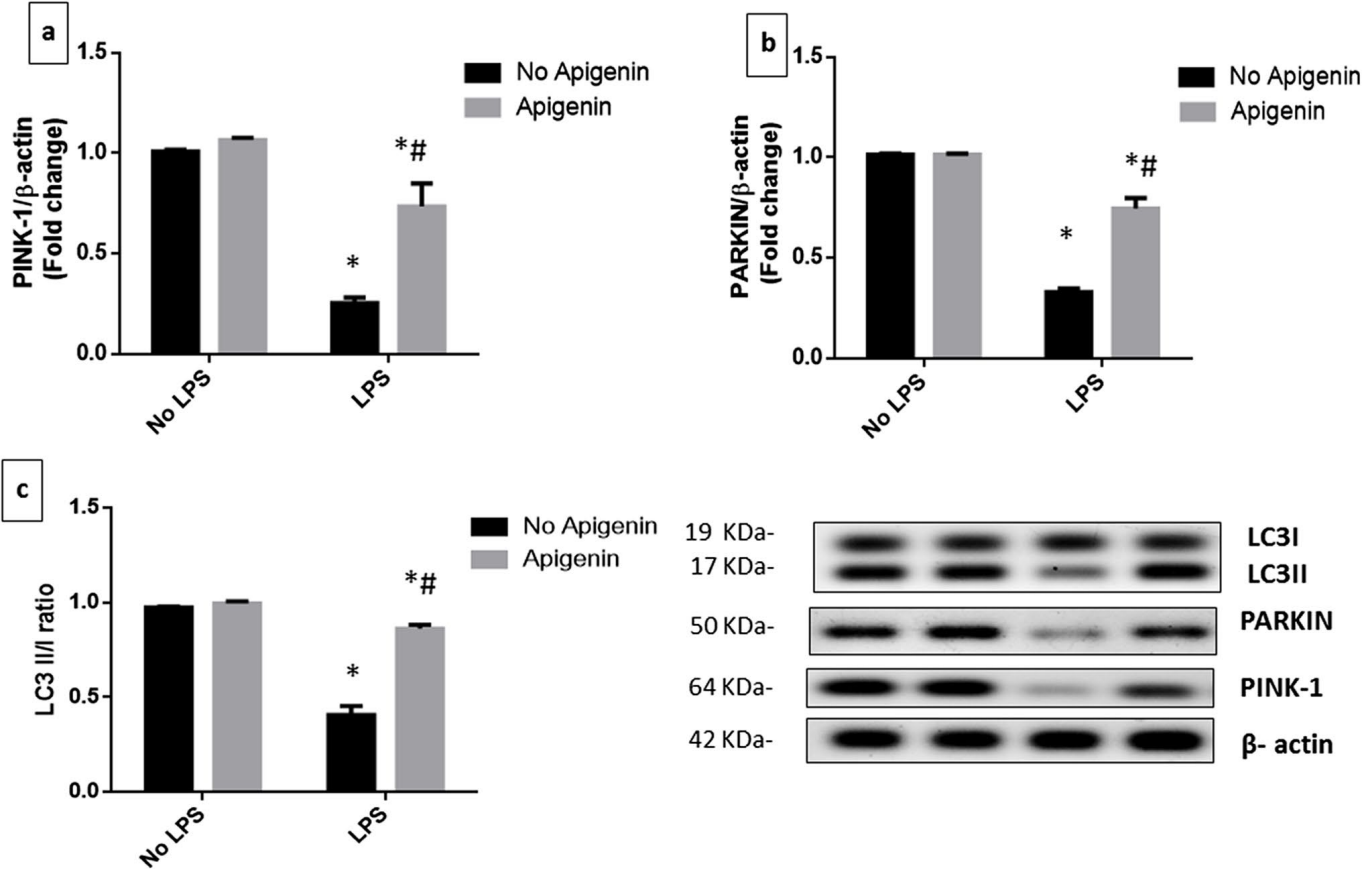
Effect of apigenin on ICV-LPS-induced alternations in mitophagy parameters. **a** PINK-1, **b** Parkin, and **c** LC3II/I. Each bar with a vertical line represents mean ± S.D (*n* = 4). Statistically significant differences (< 0.05) from the normal control group and from the ICV-LPS group are denoted by * and # respectively using two-way ANOVA followed by Tukey’s multi-comparison test

Mice which received ICV-LPS exhibited a marked reduction in PINK1 [*F* (1, 12) = 334.4, *p* < 0.0001], Parkin [*F* (1, 12) = 1171, *p* < 0.0001] expression and LC3II/I ratio [*F* (1, 12) = 722.6, *p* < 0.0001], as compared to their control counterparts. In contrast, apigenin hindered LPS-induced decline in mitophagy parameters reflected by the 2.88-, 2.26-, and 2.1-fold increase in PINK1 [*F* (1, 12) = 81.29, *p* < 0.0001], Parkin [*F* (1, 12) = 221, *p* < 0.0001], and LC3II/I ratio [*F* (1, 12) = 329.2, *p* < 0.0001], respectively related to ICV-LPS group. Interactions were recorded between the two factors (LPS and apigenin) for PINK1 [*F* (1, 12) = 51.3, *p* < 0.0001], for Parkin [*F* (1, 12) = 221, *p* < 0.0001] and for LC3II/I ratio [*F* (1, 12) = 557.8, *p* < 0.0001] (Fig. 3a–c).

### Apigenin mitigated ICV-LPS-induced changes in mitochondrial structure

**Fig. 4:**
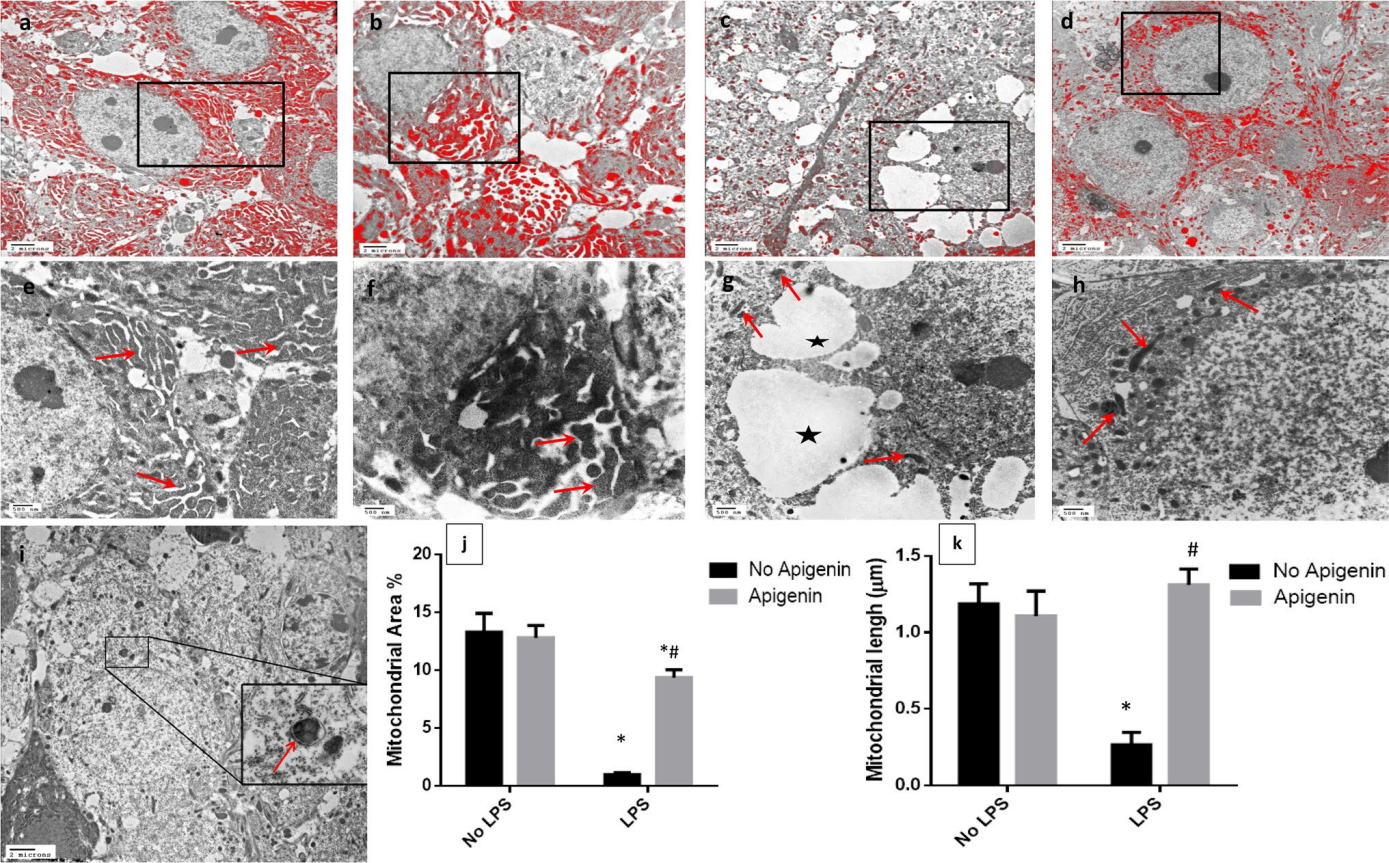
Effect of apigenin on ICV-LPS-induced alternation in mitochondrial structure. Representative colored segmented TEM demonstrating total area and density occupied by mitochondria in hippocampal pyramidal neurons with higher magnification of annotated boxes in normal control samples (**a** and **e**), apigenin control samples (**b** and **f**), ICV-LPS samples (**c** and **g**), and ICV-LPS/Apigenin samples (**d** and **h**). **i** Demonstrating autophagic membranous vacuole in a pyramidal neuron containing damaged mitochondria in ICV-LPS/Apigenin samples (boxed area). Red arrows = mitochondria, star = cytoplasmic vacuolation and reticulation. The mean of mitochondrial area percentage (**j**) and the mean mitochondrial length (**k**). Each bar with a vertical line represents the mean ± S.D (*n* = 4). Statistically significant differences (< 0.05) from the normal control group and from the ICV-LPS group are denoted by * and # respectively using two-way ANOVA followed by Tukey’s multi-comparison test

Transmission electron microscope (TEM) examination of pyramidal neurons in normal control (Fig. 4a and e) and apigenin-treated samples (Fig. 4b and f) showed the abundant presence of mitochondria which were distributed through the cell with relative mean area percentage as manually annotated and color segmented 13.3% and 12.8%, respectively. Moreover, the mean mitochondrial length was up to 1.8 microns and 1.11 microns, respectively. A significant severe mitochondrial loss was observed in ICV-LPS group samples of 92.6% compared to normal control samples [*F* (1, 12) = 231.3, *p* < 0.0001] (Fig. 4c and g) accompanied by a severe mitochondrial fragmentation and decreased mean mitochondrial length by 77.5% [*F* (1, 12) = 33.38, *p* < 0.0001] as compared with normal control samples. Contrariwise, a significant protective effect was shown in ICV-LPS/apigenin-treated mice as compared to the ICV-LPS group [*F* (1, 12) = 57.34, *p* < 0.0001] with only a 29.7% reduction in relative mitochondrial area percentage compared with normal control (Fig. 4d and h). Furthermore, minimal mitochondrial fragmentation was observed as compared to the ICV-LPS group [*F* (1, 12) = 60.74, *p* < 0.0001] with mean mitochondrial length approaching those obtained with control samples. Moreover, few records of autophagic membranous vacuole containing disrupted circular, electron-dense mitochondria showing no discernible cristae in some pyramidal neurons were observed (Fig. 4i).

Interactions were recorded between the two factors (LPS and apigenin) for mitochondrial area percentage [*F* (1, 12) = 73.7, *p* < 0.0001], and for mitochondrial length [*F* (1, 12) = 82.3, *p* < 0.0001].

### Apigenin amended alterations induced by ICV-LPS in hippocampal NAD + /NADH ratio and mitochondrial SIRT3 activity

**Fig. 5:**
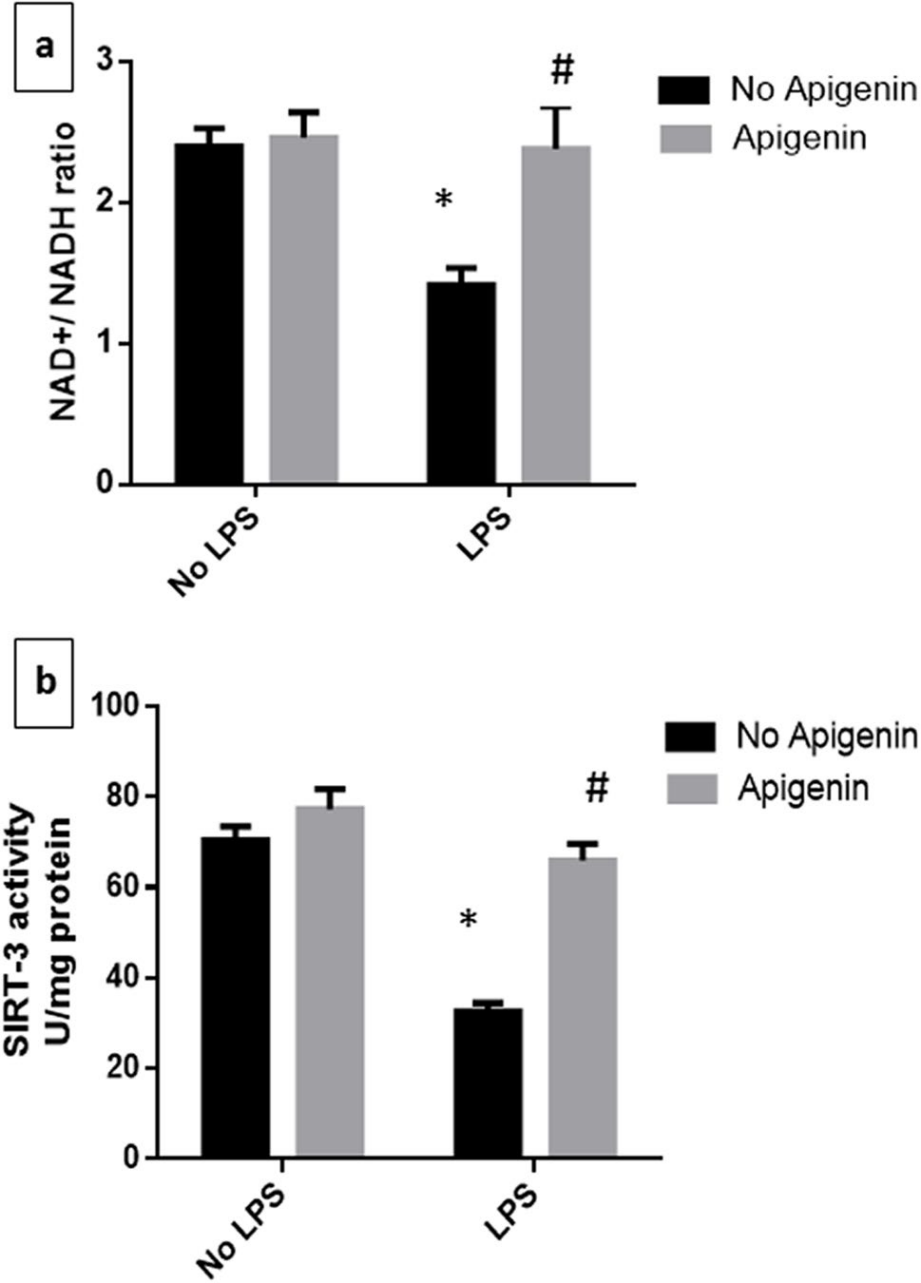
Effect of apigenin on ICV-LPS-induced changes in hippocampal **a** NAD^+^/NADH ratio and **b** mitochondrial SIRT3 activity. Each bar with a vertical line represents the mean ± S.D (*n* = 6). Statistically significant differences (< 0.05) from the normal control group and from the ICV-LPS group are denoted by * and # respectively using two-way ANOVA followed by Tukey’s multi-comparison test

As shown in Fig. 5, ICV-LPS administration resulted in a severe decline in NAD^+^/NADH ratio to 59% [*F* (1, 20) = 43.43, *p* < 0.0001] as well as mitochondrial SIRT3 activity to 46.3% [*F* (1, 20) = 320.1, *p* < 0.0001], relative to control mice. On the contrary, mice that received apigenin before LPS displayed a noticeable increase in NAD^+^/NADH ratio [*F* (1, 20) = 60.61, *p* < 0.0001] and SIRT3 activity [*F* (1, 20) = 214.1, *p* < 0.0001] reaching 1.7- and twofold the ICV-LPS group. Interactions were recorded between the two factors (LPS and apigenin) for NAD^+^/NADH [*F* (1, 20) = 31.22, *p* < 0.0001], and for SIRT3 activity [*F* (1, 20) = 92.96, *p* < 0.0001].

### Apigenin preserved hippocampal neuronal integrity and protected against ICV-LPS-induced histological and morphological alterations in mice hippocampi

**Fig. 6:**
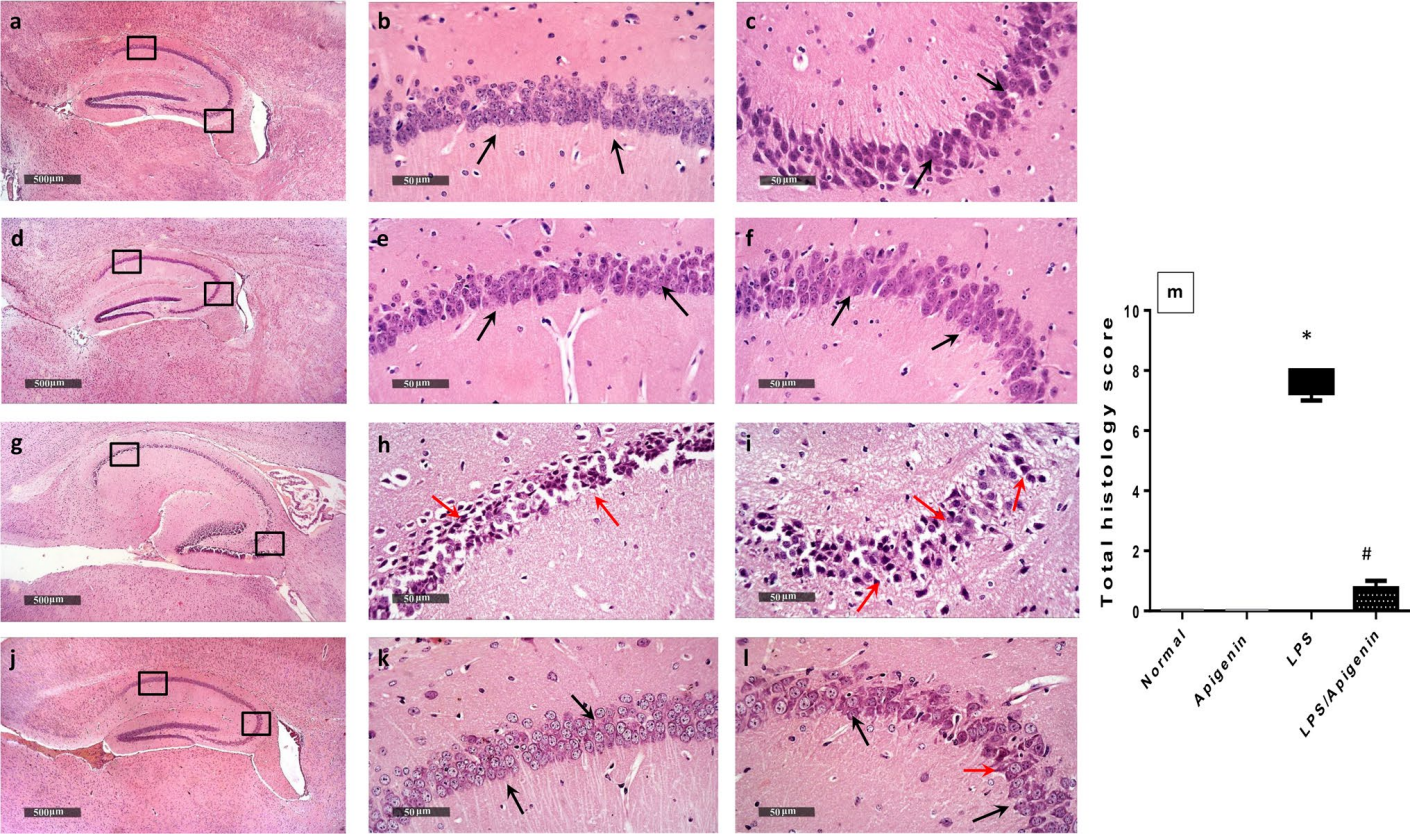
Effect of apigenin on ICV-LPS- induced histological and morphological alterations in H&E-stained mice hippocampi. Histological features of hippocampal subregions CA1 and CA3 with higher magnification of annotated boxes. Normal control samples **(a–c**), apigenin-treated samples (**d–f**), ICV-LPS model samples (**g–i**), and ICV-LPS/apigenin-treated samples (**j–l**). Total histology score (**m**), data are expressed as box plots of the median. Statistically significant differences (*p* < 0.05) from the normal control group and from the ICV-LPS group are denoted by * and #, respectively using the Kruskal–Wallis test followed by Dunn’s test

**Fig. 7:**
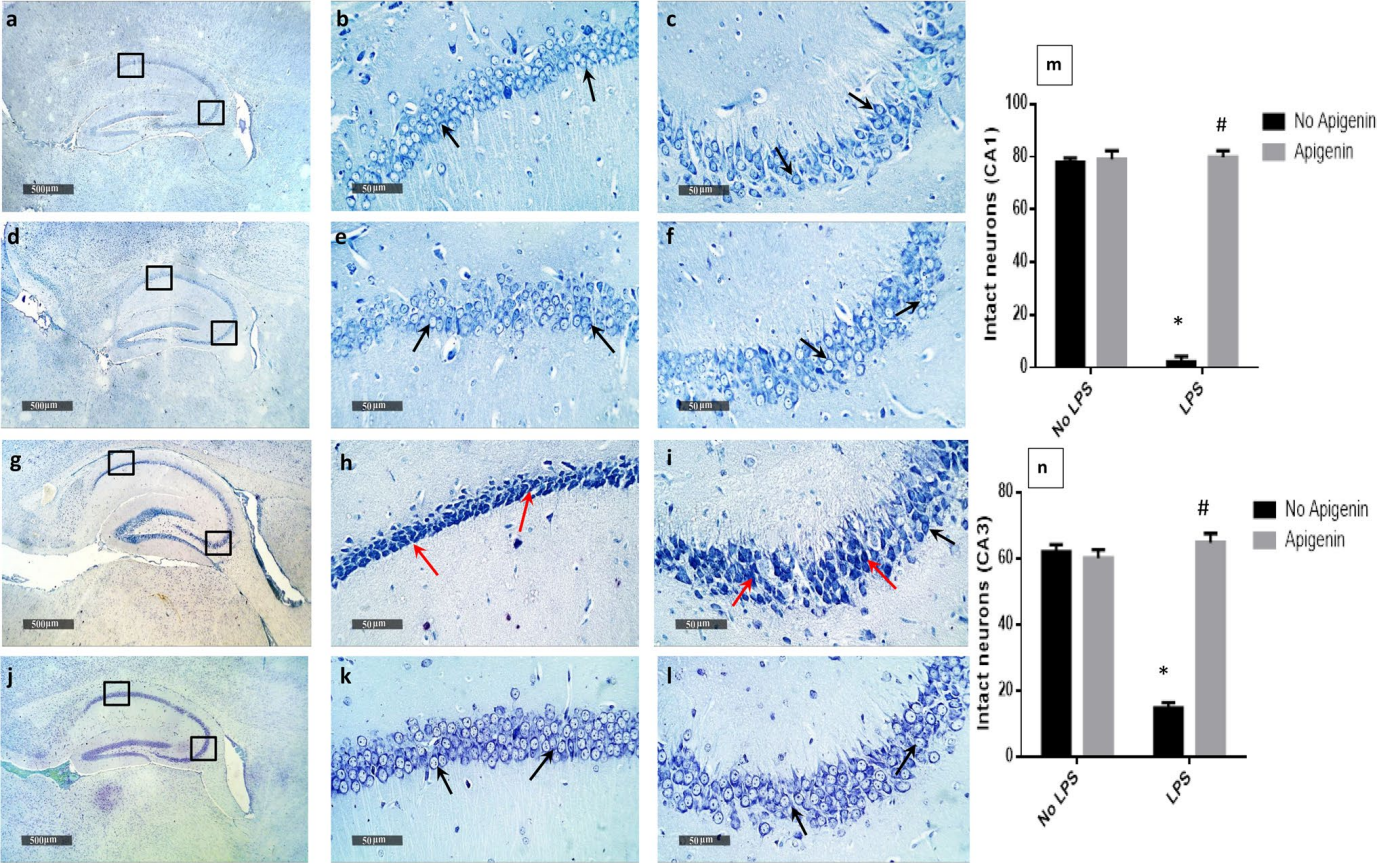
Effect of apigenin on ICV-LPS induced-hippocampal neuronal loss. Photomicrographs demonstrating toluidine blue-stained hippocampal tissue sections with higher magnification of annotated boxed CA1 and CA3 subregions in normal samples (**a–c**), apigenin control samples (**d–f**), ICV-LPS samples (**g–i**) and ICV-LPS/apigenin-treated samples (**j–l**). Black arrows point to apparent intact pyramidal neurons with intact subcellular details. Red arrows point to darkly stained and damaged perikarya with neuronal degenerative changes records. Intact neurons count in CA1 (**m**) and CA3 (**n**). Each bar with a vertical line represents the mean ± S.D (*n* = 4). Statistically significant differences (< 0.05) from the normal control group and from the ICV-LPS group are denoted by * and # respectively using two-way ANOVA followed by Tukey’s multi-comparison test

Light microscopic examination of hippocampus regions in normal control samples showed normal morphological features of hippocampal Cornu Ammonis subregions CA1, CA2, and CA3 as well as dentate gyrus region (Fig. 6a). The CA1 subregion showed an organized polymorphic layer, main cellular 5–6 cells thick pyramidal neurons middle layer with large vesicular nuclei (Fig. 6 black arrows) (mean intact pyramidal neurons count in toluidine blue-stained sections = 77 cells/field) (Fig. 7a and b) and an intact molecular layer. Intact neuropil was demonstrated with normally distributed glial cells and normal vasculatures were observed. Well-organized morphological structures were demonstrated in the CA3 sub-region with an intact polymorphic layer, main cellular pyramidal neurons middle layer 4–5 cells thick with apparent intact subcellular details (Fig. 6c black arrows) (mean intact pyramidal neurons count = 62 cells/field) (Fig. 7a and c) and a clearly intact molecular layer. Apigenin showed almost intact histological features of hippocampal subregions resembling normal control samples without abnormal cellular alteration records (Fig. 6d–f black arrows). (Mean intact pyramidal neurons count = 79 cells/field and 60 cells/field in CA1 and CA3, respectively (Fig. 7d–f). However; ICV-LPS hippocampal samples showed severe neuronal degenerative changes and loss in CA1 and CA3 subregions as compared to normal mice with many shrunken, pyknotic, and hypereosinophilic pyramidal neurons (Fig. 6g–i red arrows) with severe perineuronal edema as well as extended edema in the neuropil of polymorphic and molecular layers. The mean intact pyramidal neurons count was almost 2 cell/field and 14 cells/field in CA1 [*F* (1, 12) = 927.9, *p* < 0.0001] and CA3 [*F* (1, 12) = 73.7, *p* < 0.0001], respectively in toluidine blue-stained tissue sections (Fig. 7g–i). Obvious protective efficacy was observed by light microscopic examination in ICV-LPS/apigenin-treated samples with well-organized morphological features of hippocampal regions demonstrating many apparent intact pyramidal neurons were shown in CA1 and CA3 subregions as compared to ICV-LPS group (Fig. 6j–l black arrows) with sporadic few occasional records of neuronal degenerative changes were observed in CA3 zone (Fig. 6j–l red arrow) accompanied with intact neuropil with minimal records of edema or abnormal glial cells infiltrates. The mean intact pyramidal neuron count was 79 cells/field and 65 cells/field in CA1 [*F* (1, 12) = 1030, *p* < 0.0001] and, CA3 [F (1, 12) = 422.1, *p* < 0.0001] respectively in toluidine blue-stained tissue sections (Fig. 7j–l). Interactions were recorded between the two factors (LPS and apigenin) for intact pyramidal neuron count in CA1 ([*F* (1, 20) = 965.5, *p* < 0.0001], and CA3 [*F* (1, 20) = 495.4, *p* < 0.0001].

## Discussion

The current investigation clearly demonstrated the protective potential of apigenin against ICV-LPS bacterial endotoxin-induced hippocampal neurotoxicity in mice. Apigenin treatment robustly guarded against neuronal degenerative changes and maintained a normal count of intact neurons in different hippocampal subregions, as evidenced by histopathological examination. As a result, apigenin markedly inhibited the deleterious effect of LPS on cognitive and memory functions. Such an effect was highly reflected in the ability of mice to retrieve spatial memories and form short-term memories in MWM and Y-maze tests, respectively.

Neurons are characterized by elevated energy demands and dysregulated mitochondrial function has been strongly associated with the pathogenesis of neurotoxicity (Lee et al. 2018; Meng et al. 2019). LPS was reported to trigger extensive mitochondrial fission in neurons (Nair et al. 2019; Harland et al. 2020). Abnormal mitochondrial fragmentation is a chief mediator of mitochondrial damage via hindering oxidative phosphorylation and, consequently, ATP generation (Liu et al. 2020) . In parallel, excessive NAD^+^ exhaustion was reported in mice brains after injection with LPS (Peng et al. 2020). An aberrant NAD^+^ metabolic pathway was claimed to be one of the mechanisms responsible for LPS-induced neurotoxicity (Peng et al. 2020) and diminished mitochondrial function in neurodegenerative diseases (Lautrup et al. 2019). NAD^+^ is a crucial metabolite implicated in bioenergetics and counteracting the neurodegeneration process (Lautrup et al. 2019). Therefore, maintaining NAD^+^ levels has resulted in improved mitochondrial function and treated various mitochondrial diseases (Lee et al. 2019).

In the present study, apigenin effectively maintained a normal ratio of NAD^+^/NADH and enhanced ATP production. Concomitantly, electron microscopy revealed less mitochondrial fragmentation, where a marked increase in both mitochondrial length and area was noted in the hippocampi of apigenin-pretreated mice. In agreement with current results, Duarte et al. documented that apigenin largely balanced LPS-triggered mitochondrial dysfunction in endothelial cells by normalizing mitochondrial complex I activity (Duarte et al. 2013), whose mutation was accompanied by diminished NADH oxidation to NAD^+^ and impaired ATP production (Lee et al. 2019). Moreover, apigenin has been shown to increase NAD^+^ via upregulating, the NAD^+^ase CD38 enzyme, which is highly expressed by inflammatory cells, to amend diabetes-induced metabolic derangements and mitochondrial oxidative stress in experimental animals (Escande et al. 2013; Ogura et al. 2020).

The valuable effects of NAD^+^ may be derived from its capability of activating sirtuins to rewire metabolism and maintain mitochondrial integrity. NAD^+^ is considered the limiting factor for the activity of SIRT3 (Finley et al. 2011; Haigis and Sinclair 2010), the most important NAD-dependent histone deacetylase in the mitochondria (Finley et al. 2011). SIRT3 has a vital role in controlling mitochondrial function as metabolism, oxidative phosphorylation, electron transport, and oxidative stress (Meng et al. 2019). Its deregulation has been correlated with mitochondrial malfunction reported in Alzheimer’s disease (Lee et al. 2018). In a previous in vivo study, after receiving LPS, mice’s hippocampal SIRT3 expression was reduced. This caused significant memory loss and synaptic dysfunction and was correlated with decreased MMP, increased mitochondrial permeability transition pore opening, and increased mitochondrial apoptosis (Lyu et al. 2021). In the same context, SIRT3 overexpression attenuated the neuronal toxicity brought on by astrocytes that were activated by LPS/ interferon-gamma in a coculture of neurons and astrocytes (Lee et al. 2021). Besides its role in mitochondrial function, SIRT3 has been documented to induce gene expression of the indispensable fusion protein, MFN2 and increase the activity of OPA1 (Kincaid and Bossy-Wetzel 2013; Meng et al. 2019; Ribeiro et al. 2019). Furthermore, SIRT3 downregulation was accompanied by decreased OPA1 levels in a rat model of PD (Park et al. 2020). Noteworthy, MFN2 could induce the transcription of other mitochondrial fusion-linked genes including OPA1 to accomplish fragmented mitochondria fusion (Liu et al. 2020). The neuroprotective potential of MFN2 was previously studied in experimentally induced Parkinson’s disease (Zhao et al. 2021). In addition, Harland et al. attributed MFN2 neuroprotection against LPS-associated neurotoxicity in mice not only to the amendment of mitochondrial dysfunction and dynamic abnormalities but also to its ability to inhibit microglial activation (Harland et al. 2020). Furthermore, increased SIRT3 incites the expression of various genes implicated in mitochondrial biogenesis, especially PGC-1α to increase mitochondrial mass and, ultimately, their metabolic capacities (Fu et al. 2012; Meng et al. 2019). Interestingly, PGC-1α was reported to induce MFN2 expression to increase mitochondrial mass and number and protect dopaminergic neurons against rotenone-induced neurotoxicity (Peng et al. 2017). In the same context, diminished expression of PGC-1α and TFAM was noticed in the hippocampal tissues of AD patients (Sheng et al. 2012) and was associated with a substantial decrease in ATP production (Peng et al. 2017). In accordance with the aforementioned studies, LPS-challenged mice exhibited a decrease in PGC-1α, TFAM, OPA1, and MFN2 levels in the current study. However, the ability of apigenin to enhance SIRT3 activity secondary to restoring NAD^+^/NADH ratio was linked to an enhanced expression of these biogenesis and fusion markers which act synergically to maintain the mitochondrial area and enhance ATP synthesis as observed herein and previously (Peng et al. 2017). Coherent with these results, apigenin could incite the expression of key genes involved in mitochondrial biogenesis and ATP synthesis to attenuate muscle atrophy in aged mice (Wang et al. 2020) and prevent rat hippocampal neuronal loss induced by the amyloid fragment (Chirumbolo 2019).

In parallel, the upsurge in SIRT3 activity observed in apigenin-treated mice was associated with an increase in the expression of the fundamental mitophagy regulators, PINK1 and Parkin. Additionally, apigenin efficiently increased the LC3II/I ratio, indicating the successful formation of the autophagosome. These observations were in line with numerous studies revealing that SIRT3 could endorse PINK1/Parkin-mediated mitophagy (Das et al. 2014; Yu et al. 2017; Wang et al. 2018). In the same context, SIRT3 inhibition has decreased the expression of both PINK1 and Parkin and prevented the initiation of mitophagy. Furthermore, SIRT3 deficiency diminished the conversion of LC3I to lipid-bound LC3II, hindering the process of autophagosome formation (Kimura et al. 2009). When exposed to chronic LPS, Parkin-knockout mice experienced the loss of nigral dopaminergic neurons and fine-motor impairments. Similarly, pro-inflammatory cytokines are produced at higher levels in vitro by LPS-activated murine microglia cell line with decreased Parkin levels (Quinn et al. 2020).

While it is critical to remove damaged mitochondria via mitophagy in order to reduce the consequences of insult, it is also equally imperative to produce new mitochondria as a substitute. The PINK1/Parkin system endorses simultaneous unhealthy mitochondrial elimination along with biogenesis to prevent alterations in net mitochondrial function (Ge et al. 2020). PGC-1 α was reported to be regulated by PINK1/Parkin degradation of PARIS, a KRAB, and zinc finger protein that attaches to an insulin-responsive region in PGC-1α and triggers its transcriptional repression (Shin et al. 2011). Parkin deficiency or inactivation increases PARIS, which suppresses PGC-1α, resulting in selective neuron degeneration that can be reversed by overexpressing PGC-1α and resuming mitochondrial biogenesis (Shin et al. 2011; Stevens et al. 2015). These findings imply that the role of the boosted PINK1/Parkin system by apigenin in mitochondrial homeostasis is basically attributed to its function in mitophagy and biogenesis.

Besides its role in enhancing mitophagy and biogenesis, the PINK1/Parkin pathway has been shown to be implicated in mitochondrial dynamics regulation to pin down its effect on mitochondrial quality control (Meng et al. 2019). In Drosophila cells, PINK1 or Parkin deficiency was associated with mitochondrial elongation, whereas their upregulation resulted in mitochondrial fragmentation (Yang et al. 2008; Ziviani et al. 2010). However, numerous studies have reported inconsistent results, where inhibition of either PINK1 or Parkin in primary mouse neurons, cultured human cells, or cells from patients with PD leads to mitochondrial fragmentation. Upregulation of PINK1, Parkin, or MFN2 completely rescued such an effect, leading to mitochondrial elongation and ATP synthesis (Exner et al. 2007; Kathrin Lutz et al. 2009; Wang et al. 2011). This discrepancy has been likely attributed to the difference in the time of analysis following the silencing of PINK1 or Parkin and the cell types used. The latter studies harmonize with our results, where a well-orchestrated mitochondrial mitophagy and fusion were attained to sequester LPS-induced mitochondrial dysfunction and neurotoxicity.

In conclusion, apigenin could guard against LPS-induced neurotoxicity by preserving the NAD^+^/NADH ratio and consequently boosting the mitochondrial SIRT3 activity. In turn, SIRT3 promoted mitochondrial biogenesis and regulated quality control machinery to reduce the accumulation of injured mitochondria, either via enhancing their fusion or via promoting the degradation of unhealthy ones by mitophagy to maintain normal mitochondrial function.
